# Gene–Environment Interaction in Major Depression: Focus on Experience-Dependent Biological Systems

**DOI:** 10.3389/fpsyt.2015.00068

**Published:** 2015-05-08

**Authors:** Nicola Lopizzo, Luisella Bocchio Chiavetto, Nadia Cattane, Giona Plazzotta, Frank I. Tarazi, Carmine M. Pariante, Marco A. Riva, Annamaria Cattaneo

**Affiliations:** ^1^IRCCS Fatebenefratelli San Giovanni di Dio, Brescia, Italy; ^2^Faculty of Psychology, eCampus University, Novedrate, Como, Italy; ^3^Department of Psychiatry and Neuroscience Program, McLean Hospital, Harvard Medical School, Belmont, MA, USA; ^4^Stress, Psychiatry and Immunology Laboratory, Department of Psychological Medicine, Institute of Psychiatry, King’s College London, London, UK; ^5^Department of Pharmacological and Biomolecular Sciences, University of Milan, Milan, Italy

**Keywords:** vulnerability genes, stressful life events, DNA methylation, miRNAs, depression

## Abstract

Major depressive disorder (MDD) is a multifactorial and polygenic disorder, where multiple and partially overlapping sets of susceptibility genes interact each other and with the environment, predisposing individuals to the development of the illness. Thus, MDD results from a complex interplay of vulnerability genes and environmental factors that act cumulatively throughout individual’s lifetime. Among these environmental factors, stressful life experiences, especially those occurring early in life, have been suggested to exert a crucial impact on brain development, leading to permanent functional changes that may contribute to lifelong risk for mental health outcomes. In this review, we will discuss how genetic variants (polymorphisms, SNPs) within genes operating in neurobiological systems that mediate stress response and synaptic plasticity, can impact, by themselves, the vulnerability risk for MDD; we will also consider how this MDD risk can be further modulated when gene × environment interaction is taken into account. Finally, we will discuss the role of epigenetic mechanisms, and in particular of DNA methylation and miRNAs expression changes, in mediating the effect of the stress on the vulnerability risk to develop MDD. Taken together, we aim to underlie the role of genetic and epigenetic processes involved in stress- and neuroplasticity-related biological systems on the development of MDD after exposure to early life stress, thereby building the basis for future research and clinical interventions.

## Background

Major depressive disorder (MDD) is a leading cause of disability and disease affecting millions of individuals worldwide (1, 2). MDD includes alterations in mood, neurovegetative functions, cognition, and psychomotor activity, and it represents a major public health concern because of the short- and long-term detrimental effects to the patients and for the co-morbidities to prevalent health outcomes, such as cardiovascular disease and metabolic syndrome. The lifetime prevalence is estimated to be approximately 10–15%, and the World Health Organization has predicted that, by the year 2030, MDD will account for the 13% of the total global burden of disease replacing cardiovascular disorders (3). Due to the heterogeneity of the disorder, the diagnosis requires the presence of several symptoms including mood changes, loss of interest or pleasure, significant loss of appetite and weight, insomnia/hypersomnia, psychomotor agitation/slowing, fatigue/loss of energy, feelings of worthlessness/inappropriate guilt, inability to concentrate/indecisiveness, recurrent thoughts of death/suicide with negative consequences on work, and social relations (4, 5).

Although the etiopathogenesis of the disease is not fully understood yet, a genetic component has been recognized as exerting an important impact (6, 7). Indeed, several genetic vulnerability factors are associated with an increased risk to develop MDD, as indicated by family, twin, and adoption studies (8, 9). In particular, these studies indicate that there is a two to threefold increase in lifetime risk of developing MDD among first-degree relatives, with a heritability risk that is estimated to be around the 0.37 (95% confidence interval 0.31–0.42) based on meta-analysis data (10–12).

However, the genetic vulnerability background is not able to explain by itself the disease development. This is mainly because MDD is a complex disorder where no single gene is sufficient to cause MDD; on the contrary, each susceptibility gene contributes to a small fraction of the total genetic risk. Moreover, to make things even more complicated, MDD is known to be characterized by a complex genetic heterogeneity, meaning that multiple and partially overlapping sets of susceptibility genes interact each other and with the environment, predisposing individuals to mood disorders (7).

## Genetic Vulnerability Risk Factors for Major Depression

To date, as part of a concerted effort to understand the genetic contribution to major mental illnesses, many genetic association studies based on candidate genes or genome-wide approaches have been performed with the aim to identify susceptibility loci for MDD. However, a recent meta-analysis of genetic association studies on MDD (13) clearly concluded that candidate genes-based studies have provided only little support for the identification of vulnerability genes. Indeed, although a significant association between MDD and several genes, including apolipoprotein E (APOE) (14–16), piccolo presynaptic cytomatrix protein (PCLO) (17–19), translocase of outer mitochondrial membrane 40 homolog (TOMM40) (20, 21), guanine nucleotide binding protein (G protein) beta polypeptide 3 (GNB3) (22–24), methylenetetrahydrofolate reductase (MTHFR) (25, 26), and solute carrier family 6 (neurotransmitter transporter) member 4 (SLC6A4) (27–29) have been found in several studies, a difficulty to confirm such associations in independent samples has been also reported (30–33).

Over the past years, genome-wide association studies (GWAS), which test simultaneously common SNPs and copy number variations (CNVs), have quickly substituted the candidate genes association studies. However, they have not been able as well to clearly identify gene variants associated with MDD vulnerability (34). One of the first largest GWAS study was performed by Sullivan et al. (17) in 1,738 MDD cases and 1,802 controls, which allowed the identification of 11 signals localized to a 167 kb region overlapping the gene piccolo (PCLO). However, when they undertook validations of these SNPs in five independent samples, they were not able to replicate the findings. Subsequent GWAS and meta-analysis studies of MDD were equally unsuccessful in validating SNPs previously associated with MDD vulnerability (31, 35–37). Recently, also the largest Psychiatric GWAS Consortium study on MDD, where 9,500 cases were taken into account, has not been able to detect any significant genome-wide association (38).

The failure of GWAS analyses to provide robust evidence for loci that exceed genome-wide significance is compatible with a paradigm where MDD results from the combined action of multiple loci of small effect together with a variety of environmental factors. In line with this, when researchers started to look at the interaction between genes of vulnerability for MDD and the environment they found more robust results.

The studies above reported have been summarized in Table 1.

**Table 1:** **Genetic approach for gene–environment interaction**.

Reference	Approach	Disorder	Findings
Matsumoto et al. (28)	rs8076005 (SLC6A4)	MDD	rs8076005 AA genotype and A allele associated with changes in response rate in an antidepressant group
McFarquhar et al. (20)	rs2075650 (TOMM40)	MDD	rs2075650 G allele could be a risk factor for lifetime depression
Hu et al. (23)	C825T (GNB3)	MDD	C825T polymorphisms may be correlated with the efficacy of antidepressants in the treatment of MDD
Wang et al. (26)	Val158Met (COMT) C677T (MTHFR)	MDD	Interaction of COMT Val158Met and MTHFR C677T may be associated with cognitive function
Comasco et al. (39)	5-HTTLPR (SLC6A4) Val66Met (BDNF)	MDD	Positive association of depressive symptoms and early life events in carriers of ss/sl+Val/Val or the ll+Met
Namekawa et al. (16)	Aβ40/Aβ42 (APOE)	MDD	Serum Aβ40/Aβ42 ratio higher in patients with both early- and late-onset MDD than in controls
Verbeek et al. (18)	rs2522833 (PCLO)	MDD	rs2522833 in the PCLO gene is likely to be the causal variant in a MDD cohort
Woudstra et al. (19)	GWAS	MDD	A positive role of PCLO in symptom maintenance in MDD
Baba et al. (15)	Aβ40/Aβ42 (APOE)	MDD	Serum Aβ40/Aβ42 ratio higher in MDD patients than controls in all age groups
Hayden et al. (21)	′523 (TOMM40)	AD	Associations between ′523 and cognitive domains of memory and executive control in early-stage AD
Appel et al. (40)	rs1360780 (FKBP5)	MDD	Interactions between physical abuse and rs1360780, confirming its role in depression susceptibility
Zimmermann et al. (41)	rs1360780, rs3800373, rs9296158, rs9470080, rs4713916 (FKBP5)	Trauma	Interactions between FKBP5’s SNPs and traumatic events, with stronger effects for severe trauma
Vinberg et al. (42)	Val66Met (BDNF)	Affective dis.	Familiar risk of affective disorder and met allele associated with higher BDNF and evening cortisol levels
Aguilera et al. (43)	Val66Met (BDNF) 5-HTTLPR (SLC6A4)	MDD	Impact of childhood adversity on depressive symptoms in Met allele carriers and in S carriers
Bet et al. (44)	9β, ER22/23EK (GR)	MDD	Interaction between 22/23EK, 9β and childhood adversity resulted in an increased risk of depression
Sullivan et al. (17)	GWAS	MDD	In a genome-wide study of SNPs in MDD, 11 signals localized to a 167 kb region overlapping PCLO
Ising et al. (45)	rs4713916, rs1360780, rs3800737 (FKBP5)	Stress	Homozygous for any variants displayed an incomplete normalization of the stress cortisol secretion
Binder et al. (46)	rs9296158, rs3800373, rs1360780, rs9470080	PTSD	4 SNPs significantly interacted with the severity of child abuse to predict level of adult PTSD symptoms
Kumsta et al. (47)	ER22/23EK, N363S, BcII, 9β (GR)	Stress	Sex specific associations between GR gene polymorphisms and HPA axis responses to psychosocial stress
Cao et al. (22)	5-HTTLPR (SLC6A4) and C825T (GNB3)	MDD	Both 5-HTTLPR S and GNB3 C825T alleles had a risk of MDD higher than single 5-HTTLPR S or GNB3 825T
Dorado et al. (27)	5-HTTLPR, CYP2C9*3 (SLC6A4)	MDD	The frequency of subjects with 5-HTTLPR-S and CYP2C9*3 alleles was higher in MDD than in controls
Munafo et al. (33)	5HTTLPR (SLC6A4)	MDD	5HTT-LPR genotype significantly associated with neuroticism and lifetime major depression
Wüst et al. (48)	BcII RFLP, N363S, ER22/23EK (GR)	Stress	Impact of GR gene polymorphisms on cortisol (and ACTH) responses to psychosocial stress
Caspi et al. (49)	5-HTTLPR (SLC6A4)	MDD	After life events, 33% of individuals with an s allele, as compared to the 17% of l/l subjects became depressed
Holmes et al. (14)	rs7412 (APOE2)	MDD, AD	ApoE E2 (rs7412) allele in AD is found to be highly associated with depressive symptomatology
Arinami et al. (25)	C677T (MTHFR)	Schizo, MDD	Homozygous for the C677T allele frequently observed in schizophrenics and MDD patients

## Gene–Environment Interaction in Major Depression

Gene–environment interactions reflect a causal mechanism where one or more genetic variants and one or more environmental factors contribute to the causation of a condition in the same individual with genetic factors influencing the sensitivity to environmental exposures (50). A number of environmental factors have been found to contribute to depression vulnerability, including *in utero* exposure to infection, lack of nutrients, maternal stress, perinatal complications, social disadvantage, urban upbringing, ethnic minority status, childhood maltreatment, bullying, traumatic events, cannabis use, and exposure to stress (51–55). Among these environmental factors, stressful life experiences, especially those occurring early in life, have been suggested to exert a crucial impact on brain development leading to permanent functional changes that may contribute to lifelong risk for mental health outcomes (56–58). Indeed, during periods of heightened neural plasticity throughout development, brain regions involved in the regulation of emotion and in the mediation of the stress response appear to be particularly sensitive to the effects of stressful events. Such experience-dependent plasticity may produce altered neural circuits and maladaptive responsiveness to the environment that, ultimately, lead to an enhanced risk for depression (59).

This also suggests that genes and environment start to interact early during the development and that higher liability to psychopathology may originate when environmental challenges do occur in the pre-perinatal period. This situation may result in a variety of outcomes based on the severity of both genetic and environmental profiles for a particular disorder, and also on the presence or absence of other protective factors, which may modulate the risk for subsequent psychopathology (60). Stressful experiences during early childhood can thus significantly undermine the development of adaptive mechanisms required to deal with challenges in the adulthood and may also contribute for unhealthy lifestyles, negative interpersonal relationship, and poor health outcomes (61–63).

The mechanisms that mediate the impact of early life adversities on depression risk have been object of studies over decades. Chapman et al. (64) reported a dose–response relationship between the severity of experienced childhood adversities and the presence of a depressive episode or lifetime chronic depression. The experience of any childhood adversity has been indeed associated with an increased risk of suicide attempts in different period of life including childhood, adolescence, and also adulthood (65). In addition to maltreatment, parental loss due to death or separation or an adverse family environment characterized by poor paternal relationships or maternal overprotection has been also associated with increased risk for depressive disorders (66–69). However, it is important to mention that, although early life stressful experiences have an impact on MDD vulnerability, they do not lead to psychiatric disorders in all the exposed individuals, since the outcome is highly dependent on the individual genetic background, which, in turn, can regulate/influence the mechanisms of coping to stressful stimuli (70, 71).

On these bases, a large body of research has thus focused on identifying genetic variations that interact with early life adversities in predicting current or lifetime MDD diagnosis, or symptom severity. Here below, we discuss how genes involved in stress response and neuroplasticity interact with the environment, in particular with stressful experiences, modulating the risk for MDD.

Caspi et al. (49) were the first showing the effect of gene and environment interaction in modulating the risk for depression development. They focused on the role of a common functional 43 bp insertion/deletion polymorphism (5-HTTLPR) in the promoter region of the serotonin transporter gene (SLC6A4 or SERT). This polymorphism is known to involve either short (s) or long (l) alleles, with the short allele variant being associated with lower promoter transcriptional efficiency (59). The authors, studying the Dunedin Birth Cohort of New Zealand, showed that 5-HTTLPR interacts with stressful life events, in particular childhood maltreatment, to predict current and lifetime diagnoses of MDD as well as suicide attempts. They reported that, in the context of exposure to stressful life events, subjects’ homozygotes (s/s) or heterozygotes (s/l) for the short allele exhibited more depressive symptoms, diagnosable depression, and increased suicide behavior compared to individuals homozygous for the long allele (l). In particular, if exposed to stressful experiences during life, the 33% of individuals with an s allele, as compared to the 17% of l/l homozygotes subjects became depressed (49).

Other similar studies have been then performed to investigate the role of 5-HTTLPR short variant in increasing the vulnerability for depression development upon exposure to environmental adversity, providing both replications and failures of the original finding (72–76). Recent meta-analysis indicated, however, an absence of a clear association: indeed, although 17 positive replication studies were reported, also 8 partial replications (interaction only in females or only with one of several types of adversity) and 9 non-replications (no interaction or an interaction in the opposite direction) were indicated (74, 77, 78). The possible causes of such discrepancies could be due to potential sources of this heterogeneity, mainly stemming from methodological differences between studies (77). For example, the method of assessing early life stressful experiences, as well as the type and timing of ELS, appear to be important modifiers (75). In addition, the definition of the outcome variable seems to exert a particular influence. Furthermore, it has now been reported that the interaction between 5-HTTLPR and ELS in predicting depression is moderated by other polymorphisms in other genes (39, 79).

Another gene that may modulate the impact of stress on depression vulnerability is the brain-derived neurotrophic factor (BDNF). BDNF is a neurotrophin widely expressed in the brain where it is implicated in neuronal growth, synaptic plasticity, and neuronal survival, and it plays important roles in structural brain abnormalities observed in depressed individuals, such as reduced hippocampal volume or cognitive deficits (80–83).

A functional SNP (rs6265) within the BDNF gene promoter, which causes a valine to methionine substitution at codon 66 in the BDNF gene (Val66Met), has been shown to influence the activity of the BDNF protein (84–86). Moreover, the Met allele of the BDNF Val66Met polymorphism has been found associated with a reduced BDNF activity (87), memory impairment (84), harm avoidance (88), brain volume reduction, and has been also shown to affect intracellular trafficking (89). Moreover, individuals, which are heterozygotes or homozygotes for the Met allele, have elevated evening cortisol levels, suggesting an altered HPA axis functionality (42). Interestingly, still in the context of stress, interaction between this polymorphism and childhood sexual abuse in the prediction of adult depression has been demonstrated (43). Several studies have further provided evidence that the BDNF Val66Met polymorphism interact with ELS in predicting depression (59, 90) and a recent meta-analyses, which combined the results from 22 different studies, supported the idea that BDNF Val66Met polymorphism significantly moderates the relationship between life stress and depression (91).

The regulating effects of stress on brain and behavior are mediated by the binding of the stress hormone, cortisol, to specific receptors: the high-affinity mineralocorticoid receptors (MR), which exert tonic inhibitory effects on basal HPA axis activity, and the high-affinity glucocorticoid receptors (GR), which are critical in regulating stress responses characterized by elevated cortisol levels. The GR is kept at cytosolic level in an inactive state by the binding with FK506 binding protein 51 (FKBP5), a co-chaperone of hsp90, which prevents its translocation to the nucleus. When cortisol binds the GR-hsp90 complex, FKBP-5 acquires a reduced affinity for the complex and it is substituted by another co-chaperone named FKBP4 that, on the contrary, facilitates nuclear GR translocation leading to transcription of GR-dependent genes. An alteration in GR functionality, also known as GR resistance, is a well-characterized feature of depression and it has been associated with HPA axis hyperactivity. Given the important role of GR in regulating stress responses and the evidence for GR resistance in depression, polymorphisms within GR or FKBP5 genes have been studied as possible genetic factors for depression vulnerability. To this regard, Wüst et al. (48) have shown an impact of polymorphisms within GR gene on cortisol and ACTH responses to psychosocial stress. Specifically, 112 healthy males were studied to estimate the impact of three GR gene polymorphisms (*Bcl*I RFLP, N363S, ER22/23EK) on cortisol and ACTH responses to psychosocial stress (Trier social stress test) with the conclusion of a strong association (48).

Moreover, a strong gene × environment interaction involving functional polymorphisms within GR gene has also been shown by Bet et al. (44). In particular, the authors investigated the GR 22/23EK and 9β polymorphisms showing how they do predict the development of clinically relevant depression by interacting with environmental adversities during adolescence and childhood including war experiences, sexual abuse, parental loss, or physical illness (44). This evidence has been supported by Kumsta and colleagues, which showed a significant sex specific association between GR gene polymorphisms in 9β and *Bcl*I and HPA axis responses to psychosocial stress as well as GC sensitivity (47). These findings support the relevance of GR gene polymorphisms in HPA axis regulation and suggest that they might represent a risk factor in the development of stress-related disorders like MDD.

Also, variants within the FKBP5 gene have been shown to modulate the risk of developing MDD in relation to stressful experiences (92). For example, Appel et al. (40) reported a significant gene × environment interaction by investigating the effect of a functional SNP within FKBP5 gene (rs1360780) on depression development in more than 2,000 German people. In particular, they found that TT subjects with a history of physical abuse had an enhanced risk for depression development when compared to CC/CT individuals. This interaction between the rs1360780 and traumatic life events in predicting the onset of MDD was confirmed by another study performed in 884 adolescent and young adult individuals characterized for traumatic life events (UK environmental risk longitudinal twin study) (41). Subsequently, the same SNPs within the FKBP5 gene have been associated also with peritraumatic dissociation among injured children (93), with recovery from psychosocial stress in normal controls (45), and also with the association between childhood abuse and the development of post-traumatic stress disorder (46). Another evidence that supports the role of FKBP5 as mediator of stress exposure comes from a study where a particular FKBP5 haplotype of four SNPs (rs3800373, rs9296158, rs1360780, rs9470080) was found associated with an increased risk of suicide attempts but only in individuals with a history of childhood trauma (94).

These results, taken together, strongly support a role of the FKBP5 gene in the pathogenesis of stress-related depression, likely mediated through the influence of individual level of GR resistance and, consequently, glucocorticoid signaling.

## Epigenetic Mechanisms as Mediators of the Effect of the Environment on the Genome: Focus on DNA Methylation and miRNAs

The term “epigenetics” refers to the potentially heritable, but environmentally modifiable, regulation of genetic function and expression (95). Among epigenetic processes, DNA methylation is one of the major epigenetic processes studied in the context of early life adversities as a potential mechanism to explain the long-term effects on gene transcription (96–98). DNA methylation is a covalent modification of the cytosine residues that are located primarily at CpG dinucleotide sequences in mammals; methylation changes within promoter and enhancer regions of the gene are particularly important as they reduce the access of transcription factors to regulatory elements and promote silencing of gene expression (99). The contribution of DNA methylation has been extensively investigated especially in the context of pathologies related to exposure to stressful life events (especially those occurring early in life, ELS) including depression or post-traumatic stress disorder (73, 95, 98, 100, 101).

Studies conducted both in animal models and in humans have shown that ELS can leave persistent epigenetic marks on the genome, which can influence neurobiological substrates until adulthood (56). Indeed, early-life exposures can disrupt epigenetic programing in the brain, with long-lasting consequences for gene expression and behavior (102).

Altered DNA methylation profiles in response to ELS have been observed not only in specific candidate genes, both at central or peripheral level (103, 104), but also on a genome-wide level (105, 106). Recently, Nagy and colleagues found differentially methylated regions of DNA in astrocytes, which are glial cells specific to the CNS, of human prefrontal cortex between cases (depressed patients) and control subjects, revealing reduced methylation levels in cases (107). Indeed, emerging evidence has suggested that ELS can induce structural brain changes through epigenetic mechanisms (108). These alterations include a loss of dendritic spines and synapses, a reduced dendritic arborization together with diminished glial cells and have been widely found in the hippocampus of MDD subjects (109). Interestingly, antidepressant treatment can reverse stress-induced structural changes augmenting dendritic arborization and synaptogenesis (109).

Another quite new area in epigenetic research is represented by small RNAs, in particular microRNAs (miRNAs), which are small non-coding RNAs (20–22 nt) that play a major role in post-transcriptional regulation of gene expression. miRNAs are predicted to influence the expression of more than 60% of all the protein-coding genes through a post-transcriptional mechanism by base-pairing to target mRNAs. Generally, miRNAs inhibit protein synthesis either by repressing translation or by inducing deadenylation and degradation of target mRNAs, but were also reported to activate translation (110, 111). Individual miRNAs have the potential to target hundreds of different mRNAs, and a single gene could be modulated by several different miRNAs, thus implying a coordinate and fine-tuned expression of proteins in a cell and even, in particular, cell compartments (112, 113).

It is also known that environmental factors may modify gene expression through the regulation of miRNA synthesis (114), and there is evidence for a bilateral interaction between miRNA expression and other biological processes modulated by environment including DNA methylation or histone modifications (95, 115–117).

Examples of the effect of environmental factors on miRNAs come from preclinical data, where rats exposed to chronic stress showed an increased expression of miR-186 and miR-381 and a down-regulation of miR-709; interestingly, such alterations persisted over time as miR-709 alterations were still present also after 2 weeks of recovery from stress, suggesting that miRNAs changes can persist over time and can mediate altered stress-induced behaviors (118). Moreover, a maternal separation paradigm was able to up-regulate the expression of miR-132 and miR-124 in the prefrontal cortex of 14-day-old pups, an effect that, again, can be observed also in adult rats (119). These long-lasting modifications of miRNAs may also result from epigenetic changes since a recent human study identified miRNAs possible target of genome methylation after childhood abuse exposure (120). In this study, the authors were able to demonstrate an abuse-associated hypermethylation in 31 miRNAs in a sample of adult males exposed to childhood abuse and, at least for 6 of these miRNAs, the hypermethylated state was consistent with the hypomethylation of their gene targets (120).

Here below, we now report some examples of epigenetic processes (DNA methylation and miRNAs changes) as modulators of target genes involved in neuroplasticity, serotoninergic transmission, and GR functionality and, in particular, we will focus on the same genes we have already discussed in previous paragraphs as involved in gene × environment interaction.

The neurotrophin BDNF is a molecule highly sensible to stress, and its expression is reduced in key brain regions of animal models of depression (121–123) and also in the blood of depressed patients (124–126). In order to better understand the possible mechanisms underlying these changes, researchers investigated the role of DNA methylation within BDNF promoter regions. For example, adult rats exposed to early maltreatment showed reduced BDNF mRNA levels in the prefrontal cortex, as a result of an increase in the methylation of CpG sites within the promoter region of the BDNF exon IV. These changes persisted through adolescence and were maintained up to adulthood. Moreover, alterations in the methylation pattern at BDNF IV exon was also found in the offspring of females that had previously experienced the maltreatment regimen, indicating that these epigenetic processes can be perpetuated from one generation to the next (127).

These data have been supported by human studies. Fuchikami and collaborators examined the methylation profile of two CpG islands localized within two promoter regions (I and IV) of the BDNF gene in peripheral blood from depressed patients and controls reporting a significant difference at the promoter of exon I between the two groups (128). In another study, Kang and colleagues (129) found a significant increase of BDNF gene methylation in depressed patients with suicidal attempt history, (129). Moreover, an increased methylation of BDNF exon IV promoter was associated with decreased BDNF mRNA levels in brain Wernicke’s area from suicide victims who were affected by MDD (130).

Alterations of miRNAs expression may also contribute to BDNF dysregulation. Interestingly, Li et al. (131) showed that reduced serum BDNF levels in depressed patients was accompanied by an up-regulation of two miRNAs (miR-132, miR-182), which have been previously described to regulate BDNF (132). Moreover, a reduction of BDNF expression in the brain of animals exposed to maternal deprivation was significantly associated with an up-regulation of miR-16 levels (86).

Also, the SLC6A4 (or SERT) gene has been widely studied for changes in its epigenetic status, especially in relation to stressful life events exposure or in relation to MDD. Several *in vivo* and *in vitro* studies found site-specific methylation changes within SLC6A4 gene, which were associated with decreased levels of the SERT mRNA. Moreover, Philibert and colleagues showed, by using lymphoblast cell lines, that this effect was shown only when the 5-HTTLPR genotype was taken into account (133). Higher SERT promoter methylation status was significantly associated with childhood adversities, family history of depression, higher perceived stress, and more severe psychopathology (129). There is also evidence for reduced SERT mRNA levels in individuals exposed to maternal prenatal stress or childhood maltreatment, observed by an inverse correlation between SERT mRNA levels and the magnitude of prenatal/early adversity (56). Maternal separation has been associated with lower SERT mRNA levels in rodents and decreased SERT availability in non-human primates (134). Rhesus macaques exposed to maternal aggression displayed a reduced SERT mRNA levels in peripheral blood cells, indicating that stress-induced changes of SERT expression may not be limited to the brain (135).

SERT expression can also be influenced by specific miRNAs. Interestingly, it has been shown that miR-16, which targets BDNF, is also involved in the modulation of SERT both in human and rat tissues (136), suggesting that the interaction between BDNF and the serotonergic system may be due to the action of common regulatory miRNAs (137). Interestingly, Baudry and colleagues reported that a chronic treatment with the selective serotonin reuptake inhibitors (SSRI) fluoxetine increases miR-16 levels in serotonergic raphe nuclei, and that this increase is accompanied by a reduction of SERT expression and in BDNF modulation, suggesting a role for miR-16 in the therapeutic action of SSRI antidepressants (138).

Epigenetic mechanisms have been also proposed as linking bridges between environmental factors and genes involved in the HPA axis functionality (139). In rodents, increased maternal care, such as pup licking and grooming (LG) and arched-back nursing (ABN), has been associated with long-lasting changes in DNA methylation within the exon 17 in the promoter region of the glucocorticoid receptor GR gene. In particular, the NGFI-A binding site in the GR promoter 17 was highly methylated in the offspring of low caring mothers, resulting in reduced expression of GR exon 17.

Similar studies have been performed in humans in order to investigate alterations in the GR 1F promoter, the human homolog of the rat exon 17 NR3C1 promoter, in relationship with early life adverse events. To this regard, McGowan et al. (103) have found that the methylation levels of the GR 1F promoter were significantly increased in the hippocampus from suicide victims with a history of childhood abuse, as compared with those from suicide victims with no childhood abuse or with control samples, suggesting an effect of child abuse, independent from suicide, on GR methylation status and gene expression.

Epigenetic changes on human GR promoter as consequence of early environmental factors have also been reported by Oberlander et al. (140), which showed that prenatal exposure to maternal depressed/anxious mood led to increased methylation levels of the NR3C1 promoter in cord blood of newborns and that this methylation pattern was also associated with increased salivary cortisol in response to stress (140). Childhood maltreatment was also associated with increased methylation of NR3C1 promoter in the blood of patients affected by psychiatric disorders including MDD (139). Furthermore, the severity, the number, and the type of maltreatments positively correlated with the level of methylation (139). Interestingly, GR methylation marks might be even transmitted to subsequent generations, suggesting that the vulnerability risk of the offspring toward stress-related disorders may be related to the induction of specific epigenetic signatures (141–146).

For example, Radtke et al. (147) investigated whether intrauterine exposure to maternal stress could affect the epigenetic patterns beyond infancy. In particular, they analyzed the methylation status of the NR3C1 gene both in mothers, subjected to intimate partner violence during pregnancy, and also in their adolescent children. The authors found an increased methylation of NR3C1 promoter in children exposed to prenatal stress, confirming that epigenetic modifications could be transmitted from one generation to another and could be established already *in utero*.

Also, the GR co-chaperonine, FKBP5, has been widely investigated in term of epigenetic changes induced by ELS, because of its role in modulating the stress response and its relationship to stress-related neuropsychiatric disorders, such as MDD. For example, a transient decreased methylation status accompanied by an increased expression of FKBP5 has been shown in the hippocampus, hypothalamus, and in the blood of mice after prolonged exposure to glucocorticoids (148). Reduced methylation levels of FKBP5 gene within regions containing functional glucocorticoid responsive elements were observed also in the blood of individuals exposed to childhood abuse when compared to subjects without any childhood trauma (104). The authors suggested that changes in FKBP5 methylation might increase the differential responsiveness of FKBP5 to GR activation that, if installed during developmentally critical periods, then can remain stable over time.

Recent evidences suggest that, above to DNA methylation, also miRNAs can persistently influence HPA axis responsiveness. For example, an increase of miR-18a levels, which targets the GR, was found in the hypothalamus of a stress-sensitive rat strain, while miR-34c was found markedly up-regulated after acute and chronic stress in mice (149). Differential modifications in miRNAs expression were demonstrated in relation to normal (non-learned helplessness) versus aberrant (learned helplessness) response to a repeated shock paradigm in rats (150). It was also suggested that stress-inducible cognitive impairments could be attributable to cholinergic-mediated induction of miR-132 in hippocampus. Interestingly, excess in glucocorticoids was shown to interfere with the BDNF/miR-132 cascade (151). Another GR-targeting miRNA is miR-124a, which has been suggested to be critical for maintaining GR expression at permissive levels to neurogenesis (152).

We summarized the above-mentioned studies on epigenetics in Table 2.

**Table 2:** **Correlation between epigenetic modification, stress, and genetics**.

Reference	Gene	Epigenetic modification	Findings
Kang et al. (129)	BDNF	DNA methylation	A higher BDNF promoter methylation status was significantly associated with suicidal ideation
Li et al. (131)	BDNF	miRNA	Reverse relationship between the serum BDNF levels and the miR-132/miR-182 levels in depression
Kang et al. (129)	SLC6A4	DNA methylation	Higher SLC6A4 promoter methylation status was significantly associated with childhood adversities
Moya et al. (136)	SLC6A4	miRNA	SERT expression is regulated additionally by miR-15a as well as miR-16 in human and rat tissues
Klengel et al. (104)	FKBP5	DNA methylation	FKBP5 methylation might increase the differential responsiveness of FKBP5 to GR activation, which could remains stable over time
Fuchikami et al. (128)	BDNF	DNA methylation	DNA methylation profiles of CpG I of the BDNF gene may be a valuable diagnostic biomarker for major depression
Perroud (153)	NR3C1	DNA methylation	Childhood maltreatment associated with increased methylation of NR3C1 promoter in the blood of psychiatric patients
Radtke et al. (147)	GR	DNA methylation	Methylation status of the GR gene of adolescent children is influenced by their mother’s experience of IPV during pregnancy
Haramati et al. (149)	CRFR1	miRNA	miR-34c was further confirmed to be up-regulated after acute and chronic stressful challenge
Keller et al. (130)	BDNF	DNA methylation	BDNF promoter/exon IV is frequently hypermethylated in the Wernicke area of the postmortem brain of suicide subjects
Baudry et al. (138)	SLC6A4	miRNA	miR-16 contributes to the therapeutic action of SSRI antidepressants in monoaminergic neurons
Lee et al. (148)	FKBP5	DNA methylation	After chronic exposure to CORT, 2.4-fold increase in Fkbp5 expression and a decrease in DNA methylation
Kawashima et al. (151)	GR, BDNF	miRNA	An excess glucocorticoid exposure results in a decrease in the BDNF-dependent neuronal function via suppressing miR-132 expression
Roth et al. (127)	BDNF	DNA methylation	Early maltreatment produced changes in methylation of BDNF that caused altered BDNF gene expression in the adult prefrontal cortex
McGowan et al. (103)	NR3C1	DNA methylation	Methylation levels of the GR 1F promoter increased in the hippocampus from suicide victims with a history of childhood abuse
Cheng et al. (152)	SOX9	miRNA	miR-124-mediated repression of Sox9 is important for progression along the SVZ stem cell lineage to neurons
Oberlander et al. (140)	NR3C1	DNA methylation	Methylation status of the human NR3C1 gene in newborns is sensitive to prenatal maternal mood

Overall, these evidences suggest that a GR functionality deficit due to changes in DNA methylation or miRNAs expression could lead to long-term changes in stress hormone system regulation, to alterations of neuronal circuits and other glucocorticoid receptor responsive systems, resulting in a higher risk for the development of stress-related psychiatric disorders.

## Conclusion

Despite the relative importance of genetic risk factors in the pathogenesis of MDD, gene association studies have identified, to date, only a very small number of candidate vulnerability genes that, however, can explain little of the variance. This is known also as “missing heritability” and it could be accounted by several factors including the evidence that each susceptibility gene contributes to a small fraction of the total genetic risk for MDD, and that multiple and partially overlapping sets of susceptibility genes interact each other and with the environment, modulating the individual risk to MDD.

Among environmental factors, a prominent role has been recognized for ELS. However, not all the individuals exposed to ELS develop MDD or other psychiatric illnesses, and this is mainly because the mental outcome is dependent, once again, on the individual genetic background. Thus, the best paradigm to predict the MDD risk and MDD onset is explained by gene–environment interactions, which reflect causal mechanisms, where one or more genetic variants and one or more environmental factors contribute to the causation of a condition in the same individual with the genetic factors influencing the sensitivity to environmental exposures.

Epigenetic mechanisms, and in particular DNA methylation and miRNAs expression changes, have been widely identified as processes that could play an important role in the pathogenesis of MDD as they represent the main mediators of the effect of the environment in enhancing the vulnerability risk to develop MDD. Interestingly, epigenetic changes can be transmitted from one generation to another and this is an interesting aspect, as it could also explain the transmission of MDD vulnerability across families. In addition, ELS has been also found to act, still through epigenetic mechanism, directly on brain connections and structure by reducing the number of glial cells in the brain.

Taken together, we underlie the role of genetic and epigenetic processes involved in stress and neuroplasticity-related biological systems on development of MDD after exposure to ELS, thereby building the basis for future research and clinical interventions.

## Conflict of Interest Statement

All the authors declare that the research was conducted in the absence of any commercial or financial relationships that could be construed as a potential conflict of interest.
